# Myocardial infarction following fluorescein angiography: a case report and review of the literature

**DOI:** 10.3389/fopht.2026.1823249

**Published:** 2026-05-18

**Authors:** Maram El-Geneidy, Akhila Alapati, Radwan S. Ajlan

**Affiliations:** 1 Department of Ophthalmology, University of Kansas School of Medicine, Kansas City, KS, United States; 2 Carver College of Medicine, University of Iowa, Iowa City, IA, United States

**Keywords:** adverse reactions, coronary vasospasm, fluorescein angiography, myocardial infarction, vasculitis

## Abstract

**Introduction:**

Fluorescein angiography (FA) is a commonly utilized procedure to evaluate retinal vasculature. Although relatively safe, the procedure carries a small risk of serious life-threatening adverse reactions. The purpose of this report is to describe a case of a non-ST-elevation myocardial infarction (NSTEMI) after fluorescein angiography.

**Case presentation:**

A 76-year-old woman with a history of hypertension, hyperlipidemia, and type 2 diabetes mellitus presented to the clinic with subacute-onset bilateral vision and hearing loss. Ophthalmic exam demonstrated neovascularization of the optic disc and iris in the left eye. A recent brain magnetic resonance imaging (MRI) suggested systemic vasculitis. FA was obtained to rule out Susac syndrome and evaluate for possible vasculitic etiology of her vision loss. Approximately 10 min after the procedure, she developed acute central chest pain and hypertension. She was sent to the emergency department (ED), and workup revealed a non-ST-elevation myocardial infarction for which she was admitted. Cardiac catheterization confirmed an occlusion of the right coronary artery. She was managed medically and subsequently discharged upon stabilization.

**Conclusion:**

This case reports the extremely rare and life-threatening complication of acute myocardial infarction following FA. Possible underlying mechanisms include an allergic-mediated coronary vasospasm (aka Kounis syndrome) or anxiety-related catecholamine release in pre-existing diseased myocardium. This event underscores the critical importance of thorough patient counseling, clinical vigilance, and institutional readiness to manage acute systemic emergencies associated with this common procedure.

## 1 Introduction

Fluorescein angiography (FA) is a cornerstone diagnostic procedure for evaluating retinal vasculature, involving the intravenous injection of fluorescein sodium dye followed by retinal imaging. Although performed routinely, the procedure is associated with a well-documented spectrum of adverse reactions. The most common events are mild and transient, including nausea, vomiting, and local injection site irritation (1). The wide range in incidence of reported adverse events, from 0.83% to 21.69%, largely reflects the inclusion of these minor, self-limiting side effects (2).

In stark contrast, severe, life-threatening complications are exceptionally rare, occurring in less than 1% of cases (2). These critical events include anaphylaxis, respiratory arrest, seizures, and acute cardiovascular emergencies. Acute myocardial infarction (MI) stands out as one of the most serious of these potential complications, posing a sudden and significant challenge in the ophthalmologic setting. This report describes such a case—a rare complication of an acute MI following fluorescein angiography in a patient with suspected vasculitis—to highlight the clinical presentation and underscore the need for provider vigilance.

## 2 Case description

**Figure 1:**
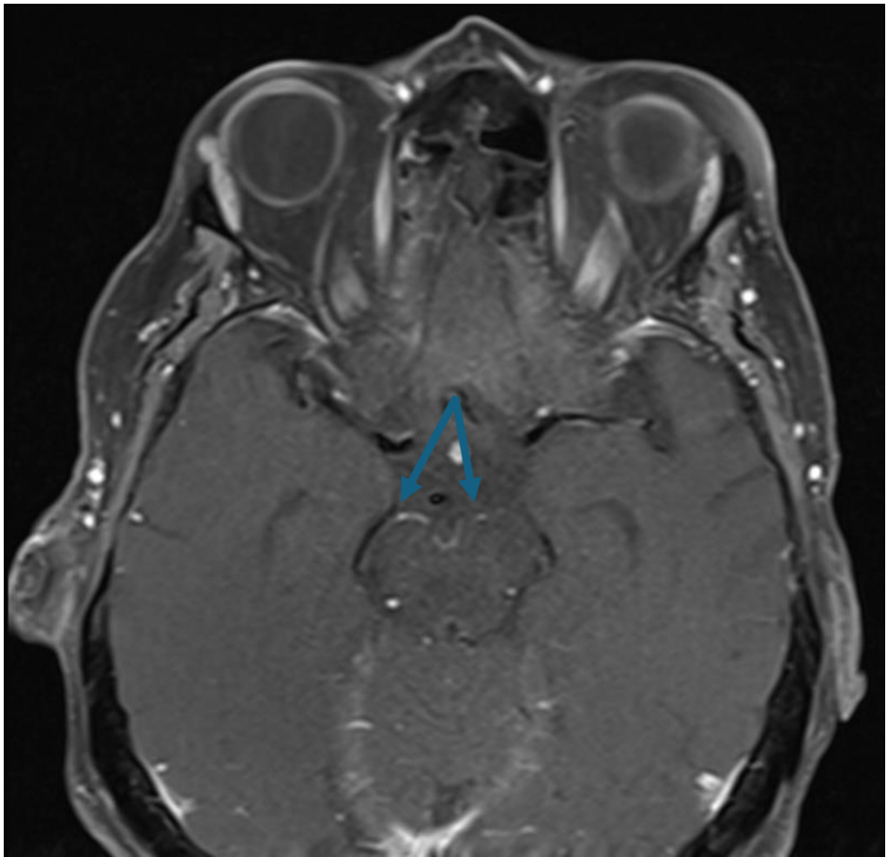
T1 MRI brain and orbits with contrast demonstrated mild enhancement at the proximal middle cerebral arteries (MCA) bilaterally, as indicated by the blue arrows. This finding was suggestive of a vasculitic process prior to the patient’s presentation to our clinic.

A 76-year-old woman with a history of hypertension, hyperlipidemia on atorvastatin, chronic obstructive pulmonary disease (COPD), hypothyroidism on levothyroxine, anxiety, and newly diagnosed type 2 diabetes mellitus was referred to the uveitis clinic for progressive, bilateral vision loss over 4 months. Her condition began with a central retinal artery occlusion (CRAO) in the left eye, followed by vision loss in the right eye 2 weeks later, which was managed at an outside clinic. She also reported a subacute history of mild hearing loss over the past 6 months and was started on 80 mg of oral prednisone for suspected autoimmune etiology. She was subsequently hospitalized for suspected autoimmune-mediated vasculitis. A magnetic resonance image (MRI) of the brain done during admission revealed mild enhancement of the proximal middle cerebral arteries (MCA) bilaterally, characteristic of vasculitis (**Figure 1**). She responded well to a 3-day course of intravenous prednisone with improvement in vision and was discharged on a tapering dose of oral prednisone (60 mg daily) and mycophenolate mofetil (500 mg daily) with scheduled follow-up with rheumatology for medical management.

On presentation to our clinic approximately 3 months after admission, she was on 35 mg of oral prednisone and 1,500 mg of mycophenolate mofetil (increased 1 month prior by rheumatology) and reported worsening vision since her discharge from the hospital. Her best-corrected visual acuity was 20/1,250 in the right eye (OD) and count fingers at 2 feet in the left eye (OS). Intraocular pressures were normal. The left eye showed trace vitreous cells, neovascularization of the optic disc and iris, vitreous hemorrhage, optic disc pallor, and peripheral vascular dropout. Given the suspicion of Susac syndrome or another underlying retinal vasculitis, a fluorescein angiogram was completed.

Approximately 10 min after returning from the imaging suite, the patient developed a central, pounding sternal chest pain and felt hot. Her blood pressure was 139/82 mmHg, and she remained alert and oriented. Emergency medical services were activated. En route to the emergency department (ED), she became nauseated and diaphoretic and was given 324 mg of aspirin. Upon arrival at the ED, her blood pressure was 172/95 mmHg with a respiratory rate of 28 breaths per minute.

The initial electrocardiogram (EKG) showed a normal sinus rhythm without ST-segment elevations. However, her serum troponin levels were noted to be uptrending, going from 6 to 28 ng/L within 2 h, and she was admitted. Repeat EKG demonstrated dynamic T-wave changes, and echocardiogram revealed an ejection fraction of 60% with areas of hypokinesis in the basal to mid inferior and inferolateral wall segments. Due to persistent chest pain, dynamic T-wave changes on repeat EKG, and areas of hypokinesis on an echocardiogram, she underwent cardiac catheterization. The procedure revealed a significant occlusion in the right coronary artery, confirming a diagnosis of non-ST-elevation myocardial infarction (NSTEMI). She was managed medically and discharged on dual antiplatelet therapy (DAPT) on 81 mg of aspirin and 75 mg of clopidogrel, 50 mg of metoprolol extended-release, and 10 mg of ezetimibe. A review of the patient’s medical record revealed prior exposure to iodinated and gadolinium-based contrast dyes without any issues. This cardiac event was documented in the patient’s medical record and listed as a reaction to fluorescein dye.

**Figure 2:**
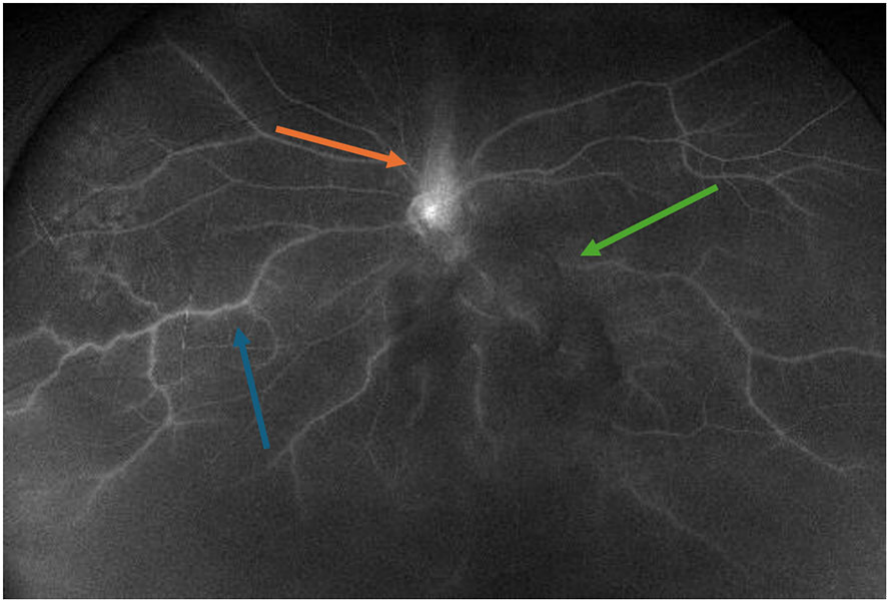
Fluorescein angiography of the left eye demonstrated vasculitis (blue arrow) and optic nerve leakage consistent with neovascularization (orange arrow). There was media opacity corresponding to vitreous hemorrhage (green arrow).

The results of the FA, reviewed after the acute event, revealed prominent vasculitis and optic nerve leakage in the left eye but did not show classic features of Susac syndrome (**Figure 2**). In light of the neovascularization of the optic nerve head and iris, she received panretinal photocoagulation (PRP) in the left eye 2 months after the acute cardiovascular event. The use of anti-vascular endothelial growth factor (VEGF) injections was deferred due to the recent NSTEMI. She was started on intravenous immunoglobulin (IVIG) 2 g/kg over 5 days as a loading dose for her vasculitis approximately 1 month after the acute cardiovascular event.

At her 3-month follow-up, the examination was stable. Subsequent visits showed regression of her iris neovascularization and improvement in the vitreous hemorrhage. Further imaging with FA was contraindicated due to her cardiac event, and a second PRP session was deferred due to poor visualization, necessitating continued clinical monitoring. She was tapered off of prednisone, and her vision remained stable on monthly IVIG 0.4 mg/kg and mycophenolate mofetil 500 mg twice daily.

## 3 Discussion

This case adds to the limited body of evidence documenting acute MI as a rare but severe complication of fluorescein angiography. To the best of our knowledge, this is the sixth in-depth reported case of myocardial infarction following FA and the only one occurring in a patient with suspected vasculitis. Our patient, with known dyslipidemia and active vasculitis, developed an NSTEMI shortly after the procedure. This event highlights the complex interplay between pre-existing cardiovascular risk factors, procedural triggers, and potential hypersensitivity reactions.

While FA is performed millions of times per year with a strong safety profile, the incidence of MI has been reported to be between 0.01% and 0.15% (2). There have been approximately 12 previously reported cases of MI after FA between the 1970s and 1990s. Nearly all reported cases involved patients with significant cardiovascular risk factors or pre-existing cardiac disease, including ischemic cardiomyopathy, arterial hypertension, diabetes mellitus, and peripheral vascular disease, establishing a pattern that continues today (3–7). The precise mechanism linking fluorescein dye to MI remains debated, as studies have not shown a direct correlation between the dye and EKG changes (8).

In one of the first documented cases of MI following FA, a 64-year-old woman with a prior history of hypertension, diabetes mellitus, and peripheral vascular disease status post-carotid endarterectomy had FA for subacute vision loss. She developed abdominal pain approximately 2 min after fluorescein injection and retrosternal chest pain radiating to the jaw within 5 min of fluorescein injection. EKG revealed ST-segment elevation. She was managed medically and discharged upon stabilization (3). Another report describes a 68-year-old man with a history of vascular disease status after right femoral endarterectomy and inferior MI who presented with acute vision loss. The patient had an FA to evaluate for macular changes and developed dyspnea and diaphoresis 30 min after fluorescein injection. EKG changes and cardiac enzymes revealed an acute anteroseptal MI. The patient was stabilized and discharged after 2 weeks (6).

McAllister describes a case of a 54-year-old man admitted for hypertension who received an FA during admission to evaluate for hypertensive retinopathy. The patient developed chest pain, diaphoresis, and severe hypertension within 30 min of the procedure. He was managed medically and discharged 15 days later (7). Cunningham et al. describe one of the first reported deaths from MI following FA in a 61-year-old man with a history of chronic stable angina, coronary artery disease, hypertension, and diabetes mellitus. This patient also had a prior history of oliguric renal failure requiring hemodialysis following coronary angiography (4). In one of the first cases documented by autopsy, a 75-year-old man with a significant cardiovascular history developed sudden chest pain immediately after fluorescein dye injection and ultimately passed away. Autopsy revealed right coronary artery outlet obstruction with fibrin–platelet thrombus and complete distal obstruction (5).

In all these previously reported cases, the patients had pre-existing cardiovascular disease or risk factors for cardiovascular disease, like coronary artery disease, peripheral vascular disease, hypertension, and diabetes mellitus, similar to the patient in our case who had pre-existing hypertension, hyperlipidemia, and diabetes mellitus. Although the exact causation between these events cannot be determined, it is possible that the diseased myocardium of these patients was more sensitive to anxiety-related catecholamine release, leading to increased downstream myocardial oxygen demands. This is a hypothesis for patients with baseline cardiovascular risk factors or disease who develop MI after FA. One report suggests that phenylephrine-dilating drops may also increase myocardial oxygen demand (6). Another suggested mechanism is the direct vasospastic effect from the dye, although this has not been evaluated or supported in the previously described cases. Our case is the first to be documented in a patient with suspected vasculitis, underscoring the potential role for vascular inflammation to predispose the patient to myocardial ischemia from coronary vasospasm.

Another compelling hypothesis is the phenomenon of “Kounis syndrome” or “allergic acute coronary syndrome.” This syndrome proposes that a hypersensitivity reaction to an antigen—in this case, the fluorescein dye—triggers the activation and degranulation of mast cells (11). The release of inflammatory mediators like tryptase and histamine can induce intense coronary vasospasm and plaque rupture, leading to acute myocardial ischemia (10, 11). Cianci et al. report a case of an 80-year-old male who developed diaphoresis and dizziness 30 min after FA. He lost consciousness and ultimately died from cardiac arrest. Post-mortem analysis revealed coronary atherosclerosis with eccentric plaques partially obstructing the lumen and areas of scattered mast cells immunopositive for anti-tryptase in the myocardial perivascular areas (9). This mechanism is particularly dangerous in patients with underlying coronary atherosclerosis, where even a partial obstruction can become critical during a vasospastic event. Our patient had suspected active vasculitis, potentially predisposing her to such an inflammatory and vasospastic response. She may have had some pre-existing coronary atherosclerosis from her hypertension, diabetes, and hyperlipidemia, which would make her more susceptible to myocardial ischemia from a vasospastic event.

The potential for an allergic-mediated MI underscores the need for meticulous patient evaluation and emergency preparedness. While a history of cardiovascular disease is a clear risk factor, this case suggests that systemic inflammatory conditions like vasculitis may also heighten patient vulnerability. Although this patient did not have reactions to previous gadolinium or iodinated contrast agents, the fluorescein agent used in fluorescein angiography may have triggered a specific antigenic response leading to coronary vasospasm. Thus, it is imperative that facilities performing FA have trained teams equipped to manage life-threatening emergencies and know when to seek immediate help (12, 13).

In conclusion, fluorescein angiography remains a vital diagnostic tool in ophthalmology. This case of NSTEMI in a patient with active vasculitis reinforces that the procedure carries rare but potentially profound risks. The likely mechanism involves an allergic-mediated coronary vasospasm (Kounis syndrome), which can precipitate an acute coronary event in susceptible individuals. This report adds to the limited body of literature reporting myocardial infarction after FA and emphasizes the importance of counseling patients on potential risks, maintaining a high index of suspicion for post-procedural cardiovascular complications, and ensuring that all staff and facilities are prepared to manage such time-sensitive medical emergencies.

## 4 Patient perspective

Upon discharge from the hospital, the patient reported she had anxiety prior to the fluorescein angiography test. After the acute cardiovascular event, she remains apprehensive about receiving any form of imaging that involves contrast dye.

## Data Availability

The original contributions presented in the study are included in the article/supplementary material. Further inquiries can be directed to the corresponding author.
